# Assessing the Feasibility of Rural Residency Training for Licensed Naturopathic Physicians in the Northwest: A Qualitative Study

**DOI:** 10.1089/imr.2022.0089

**Published:** 2023-06-23

**Authors:** Luciano Garofalo, Thomas Bell, Gena Poling, Davis Patterson

**Affiliations:** ^1^ National University of Natural Medicine, Portland, OR, USA.; ^2^ Department of Child, Family, and Population Health Nursing, University of Washington, Seattle, WA, USA.; ^3^ Bastyr University School of Naturopathic Medicine, Kenmore, WA, USA.; ^4^ Department of Family Medicine, University of Washington School of Medicine, Seattle, WA, USA.

**Keywords:** naturopathic, residency, rural, primary care, underserved, education, qualitative

## Abstract

**Objectives:**

Naturopathic physicians (ND) are uniquely situated to address areas of unmet health care need as primary care providers (PCPs). In several states, NDs have a broad scope of practice and are licensed as independent practitioners regardless of residency training. However, with a larger role in the health care system, the need for post-graduate medical training becomes more important for clinical success and patient safety. Our study aimed at assessing the feasibility of developing residencies for licensed NDs in rural federally qualified health centers (FQHCs) of Oregon and Washington.

**Methods:**

We conducted interviews with leadership from a convenience sample of eight FQHCs. Six centers were rural, two of which already employed NDs. Two urban centers that employed NDs as PCPs were included for their valuable insights related to study design. Two investigators independently reviewed and coded site visit notes for prominent themes through inductive reasoning analysis.

**Results:**

Consensus was met identifying the following themes: onboarding and mentorship; diversity of clinical training; financial structure; length of residency; and addressing health care needs in the community. We identified several opportunities for the development of primary care residencies for NDs, including the need for PCPs in rural communities, the ability of NDs to manage chronic pain with prescription drugs, and the prevention of morbidity from complex conditions such as diabetes and cardiovascular disease. Potential barriers to residency development include lack of Medicare reimbursement, mixed awareness of the ND scope of practice, and scarcity of dedicated mentors.

**Conclusion:**

These results may serve as guideposts for the future development of naturopathic residencies in rural community health centers.

*What already is known about this topic:* Naturopathic physicians (NDs) play a steadily increasing role in the primary care workforce in regions where they are licensed accordingly, but primarily in the urban setting. Most naturopathic residencies in the United States are based in urban and private clinics. The NDs can effectively treat the most common conditions seen in rural Federally Qualified Health Centers (FQHCs), but more appropriate postgraduate clinical training for NDs is needed to effectively serve in this setting.

*What this paper adds:* This study is the first to explore the potential expansion of naturopathic residencies into the rural primary care setting, and it provides a preliminary description of the current role NDs that serve in rural primary care in the United States.

## Introduction

Naturopathic physicians (NDs) are increasingly integrated into the primary care workforce in the western United States, especially in Oregon and Washington where two of the five nationally accredited doctoral programs in naturopathic medicine exist.^1–3^ Research demonstrates that NDs are effective at managing cardiovascular disease,^4^ mental illness,^5,6^ diabetes,^7^ cystitis,^8^ and back pain,^9^ among the most common conditions seen in rural primary care practices.^10,11^ In addition, complementary and alternative medicine is popular in rural areas but mostly self-administered rather than delivered by trained professionals.^12^

The NDs are trained in holistic primary care through a standardized 4-year curriculum centered on biomedical physiology and diagnostics. Naturopathic medicine is characterized by its “therapeutic order,” a conceptual guide to patient treatment that aims at optimizing therapeutic benefit and mitigate invasiveness. Naturopathic medicine not only emphasizes non-pharmacologic interventions such as lifestyle, stress management, diet, and botanicals, but also includes outpatient pharmacy, minor surgery, and referrals for specialty care when needed.^13^

Naturopathic medical students learn evidence-based medicine and critical appraisal of the scientific evidence for conventional and non-conventional treatment modalities.^14,15^ Teaching clinic networks attached to the naturopathic schools in Oregon and Washington provide primary and complementary care to Medicaid recipients and the uninsured.^16^

The NDs can address areas of unmet health care need in states where they are licensed with a broad scope of practice and are eligible for reimbursement by Medicaid and major health insurance plans. Many private clinics in such states (e.g., Oregon, Washington) are staffed by NDs who work as primary care providers (PCPs). The NDs currently work in rural Federally Qualified Health Centers (FQHCs) in these states as PCPs, in some cases incentivized to work with underserved populations through tuition reimbursement and loan repayment.^17,18^

Residency training is not required for NDs to be independent practitioners in most states; however, with a larger role in the health care system, post-graduate residency training is important for clinical success and patient safety. The Council on Naturopathic Medical Education (CNME), which accredits naturopathic schools and residency programs, strongly recommends residency training for naturopathic graduates, especially those entering the primary care workforce.^15^ In rural settings, with fewer resources and longer distances to transport emergency patients, residency training for medical providers is especially critical.^19^

Nearly all CNME-accredited naturopathic residency sites are in urban centers and private clinics that are ill-suited to prepare NDs for rural primary care practice. Rural communities are often hit hardest with primary care shortages.^20^ Because of the vital training that rural FQHCs can provide residents, NDs may be more likely to stay in rural practice if they lived and worked in this setting as residents.^21^ Like other types of physicians, it seems likely that NDs could better address the rural primary care workforce shortage if they had access to residency positions in rural FQHCs. This study aimed at exploring the feasibility of developing residency positions for licensed NDs in rural FQHCs of Oregon and Washington.

## Methods

Using a grounded theory approach, we conducted interviews with a convenience sample of administrators and lead clinicians at rural FQHCs in Oregon and Washington. The study was deemed exempt from formal review by the Bastyr University Institutional Review Board. All interviewees gave informed consent.

### Participants and data collection

We contacted 21 FQHCs in the western, central, and eastern parts of each state by phone and/or e-mail requesting interviews. All but two sites were rural (in non-metropolitan areas); non-rural sites were included, because they offered valuable information as health centers that had successfully integrated NDs as PCPs. County-wide demographics where each site is located are summarized in Table 1. We targeted rural FQHCs that had demonstrated capacity for hosting learners (residencies, clerkships, etc.). Interviews with administrators and clinicians, individually or in groups, aimed at generating a diversity of perspectives relevant to a medical residency.

**Table 1.** Demographics of County-Wide Population Where Participating Sites Are Located

	Rural 1	Rural 2	Rural 3	Rural 4	Rural 5	Rural 6	Urban 1	Urban 2
Hispanic or Latino	10.8%	63.6%	10.4%	19.5%	3.6%	50.7%	9.9%	10.7%
Race								
American Indian and Alaska Native	1.1%	2.1%	5.1%	11.6%	5.8%	5.1%	1.2%	0.8%
Asian	1.0%	0.7%	1.4%	0.7%	0.6%	1.2%	2.5%	19.9%
Black or African American	0.3%	0.2%	1.4%	0.4%	0.3%	0.8%	1.2%	6.7%
Native Hawaiian and other Pacific Islander	0.3%	0.1%	0.2%	0.1%	0.2%	0.1%	0.3%	0.9%
White	82.6%	44.5%	78.1%	65.6%	84.6%	48.5%	80.7%	56.1%
Other race	5.1%	35.3%	4.8%	11.8%	1.3%	29.2%	3.9%	5.2%
Two or more races	9.6%	17.1%	9.0%	9.8%	7.1%	15.1%	10.1%	10.4%
Household income below poverty level	12.7%	17.9%	10.1%	16.0%	12.9%	13.1%	13.0%	9.1%

Source: US Census Bureau, 2020 Census Redistricting Data, Retrieved March 29, 2023 https://data.census.gov/table

Interview questions explored the feasibility of building residencies for NDs in rural FQHCs, including clinic interest, community need, financials, and licensing. One or two investigators (T.B., G.P., L.G.) conducted interviews lasting 60–90 min in person except one, conducted via videoconference. Interviews were semi-structured, using pointed and open-ended questions relative to either the administrative or clinical role of the interviewee.

Interviewers asked follow-up questions when needed to gain clarity or flesh out previously unheard themes/subthemes. Because of the small-scale and exploratory nature of this study, detailed interview notes were typed into a Google shared document in lieu of audio-recordings to streamline coding of main themes. Interviewers also observed clinicians at four rural sites to learn about the clinic structure and patient population, and some notes were gleaned from these experiences. The study team met regularly during data collection to review interview notes, update interview questions, and determine when saturation of concepts was reached.

### Analysis

Two investigators (L.G., T.B.) independently reviewed and coded site visit notes for important themes using deductive and inductive reasoning analysis.^22^ Saturation was not assessed due to the exploratory aim of this project. These two investigators met to discuss and revise themes until consensus was reached. From these themes, we derived a list of recommendations for a potential residency program for NDs in rural primary care.

## Results

Table 1 contains the demographics for each county where participating health centers were located. A total of 16 personnel at 6 rural FQHCs and 2 urban FQHCs participated in interviews—10 administrators in a variety of roles (chief executive officer, clinic manager, senior recruiter, etc.), 5 NDs, and 1 chief medical officer. Five of the eight participating sites employed NDs at the time of the study, including the two non-rural sites.

We identified six thematic domains regarding the feasibility of rural primary care residencies for NDs, outlined in Table 2: (1) addressing clear community health care needs; (2) providing appropriate onboarding and well-structured mentorship; (3) ensuring additional clinical training; (4) establishing appropriate residency length; (5) developing financial support; and (6) promoting naturopathic primary care to rural FQHCs.

**Table 2.** Thematic Domains Relating to the Feasibility of Rural Residencies for Naturopathic Doctors, with Subthemes and Illustrative Quotes

1. Addressing clear community health care needs
NDs would need to manage complex patients without the resources of an urban medical center.
“[Providers need] a well-rounded education and bird's-eye view of the scope of healthcare.”
Some communities valued non-pharmacologic treatment and holistic approach of NDs
“The window of opportunity is stronger among rural communities because the culture of many rural populations is much closer to truly relying on nature… Many of these communities come from rural areas of impoverished countries where self-reliance and self-care was a necessity… This has cemented a profound confidence and trust in natural forms of healing as well as a healthy distrust of conventional medicine.”
Shortfalls of ND scope and training include inpatient, obstetrics, and higher intervention emergency services
2. Providing appropriate onboarding and well-structured mentorship
Onboarding of NDs would have to be iterative and dynamic, facilitating a shared confidence in the ND's patient care abilities.
“How comfortable do you feel and how comfortable do your colleagues feel? […] Nobody is safe and happy until you're feeling good about the work.”
Capacity for dedicated mentors may be higher at larger centers, but may also come with a “pecking order” of trainees
Mentorship of NDs by conventional providers would rely on an integration of clinical cultures
3. Ensuring additional clinical training
Naturopathic education should ensure NDs are prepared to work in a high-volume setting with a medically underserved population.
“Naturopathic schools need to get on board with their role in training physicians. They should teach standards of care, and NDs should be held to the same standard.”
Inpatient and emergency medicine rotations were considered high-yield experiences to prepare NDs for rural primary care.
“[Our] conventional colleagues are born in a system. You have to understand how a system works.”
4. Establishing appropriate residency length
Three years could offer the greatest benefit to rural clinics by reducing cost and improving provider retention.
“Three years is probably overkill for our scope of practice… Two years is the sweet spot.”
5. Developing financial support
Naturopathic residencies would best be funded through multiple revenue streams, such as insurance reimbursements, public/private grants, and naturopathic institutional sponsorships.
“Once you are over the hump ND residents should be revenue generators comparable to anyone else.”
Lack of Medicare reimbursement is a significant barrier to naturopathic residencies in rural FQHCs.
“[Patients who turn 65] graduate out of Dr.—-‘s office… [They] cannot pay for any services, but they want to… It's not fair, but it is what it is.”
6. Promoting naturopathic primary care to rural FQHCs
There is a variety of awareness of naturopathic training and scope of practice between FQHCs and their respective communities.
Centers with leadership that embraces complementary and integrative medicine would be more likely to “buy-in” to naturopathic medicine.
“… the broad concepts of whole person health, social determinants of health, mind-body medicine, and adverse childhood experiences become a bigger part of our narrative about ‘health’ vs. ‘healthcare.’”
More professional partnerships between NDs and rural FQHCs are likely to generate more residency and PCP positions for NDs

FQHCs, federally qualified health centers; ND, naturopathic physicians.

### Addressing clear community health care needs

Rural sites needed PCPs who could manage complex patients without the resources of an urban medical center—providers with “a well-rounded education and bird's-eye view of the scope of healthcare.” The ND skills considered valuable to a rural community included non-pharmacologic treatment of chronic pain and holistic management of other conditions with high morbidity (e.g., diabetes).

A few interviewees noted that NDs can provide high-quality patient-centered care because their communities valued naturopathic philosophy, such as the prioritization of diet and natural therapies when indicated: “The window of opportunity is stronger among rural communities because the culture of many rural populations is much closer to truly relying on nature.”^12^ One administrator from a site with a large Hispanic community reported their ND providers were popular for this reason:

*Many of these communities come from rural areas of impoverished countries where self-reliance and self-care was a necessity, and a great deal of knowledge has been passed down from generations to generations. This has cemented a profound confidence and trust in natural forms of healing as well as a healthy distrust of conventional medicine.*

A few interviewees from clinics without NDs were unsure whether NDs would be accepted as suitable PCPs by their patient population. Others noted that NDs could not fill some needs that rural PCPs typically address because they are outside the scope of ND practice, such as inpatient care, obstetrics, and some emergency medicine duties.

### Providing appropriate onboarding and well-structured mentorship

All interviewees thought the onboarding process for a naturopathic resident, as with any new provider, would need to be iterative and dynamic. Interviewees commonly recommended blocked scheduling and frequent meetings for new providers until the ND and their colleagues had a shared confidence in the ND's patient care abilities: “How comfortable do you feel and how comfortable do your colleagues feel? […] Nobody is safe and happy until you're feeling good about the work.” In addition, interviewees thought that ND residents would eventually need to manage a patient load similar to other providers at their clinic, citing reasons such as harmony in the workplace, financials, and high demands of patient care.

Capacity for mentorship in a potential naturopathic residency was generally regarded as a challenge for rural sites. Interviewees questioned whether well-established clinicians would have time left for a naturopathic resident after patient care and mentoring other learners. Interviewees at some larger sites reported more capacity for mentorship than those at smaller sites. Two sites indicated the importance of building mentorship structures into clinic systems, rather than merely volunteer mentorship.

Some administrators and NDs posited that functional mentorship would rely on integration of clinical cultures. Conventional medicine clinicians and naturopathic doctors/residents need a strong working relationship to successfully share patient management. For the sake of adequate, degree-specific training for ND residents, interviewees identified a need for mentors with a deeper understanding of the naturopathic clinical paradigm and a preference for well-established NDs at the same center.

### Ensuring additional clinical training

Several ND interviewees acknowledged shortcomings in their naturopathic education for working with a medically underserved population. “Naturopathic schools need to get on board with their role in training physicians. They should teach standards of care, and NDs should be held to the same standard.” One ND at a rural FQHC who had graduated less than 5 years earlier reported having an easier transition, which he attributed to more recent reforms in naturopathic education that improved training in primary care and evidence-based medicine.^14^

This ND's administrator vouched for his preparedness: “[ND] was suturing people within the first two or three months he was here… [he] is a community favorite.” Nevertheless, most interviewees recommended that more robust clinical training during undergraduate medical education would better prepare NDs for the transition to primary care, especially in the rural setting where providers have less support in managing more acute and complex patient conditions. Although inpatient care is outside of the naturopathic scope of practice, NDs considered inpatient and emergency medicine experiences to be the most high-yield in preparation for primary care because of the acuity of care required in these settings.

The NDs also described discord between their undergraduate clinical training, where patient visits average 60 min, versus the typical primary care visit of 20 min length. The naturopathic clinical approach favors long, detailed patient intakes to inform a holistic treatment plan, but NDs recognized this was not always practical in a busy FQHC. One ND who worked with low-income patients said she had to “be a doctor first and ND second … you cannot pursue naturopathic medicine in the same way; there is less room for the ideals.”

To this point, another ND stated: “Our doctors need to learn some basic boundaries around what is covered in one appointment. I don't need to cover everything in a visit for UTI; that visit should be five minutes.” Some NDs thought that the workflow of a high-volume FQHC would be a bigger adjustment for naturopathic residents than for Doctor of Medicine/Doctor of Osteopathy (MD/DO) residents.

### Establishing appropriate residency length

All interviewees thought that a rural naturopathic primary care residency should last at least 2 years, with most interviewees preferring three. Some suggested the conventional model of an initial intern year, with subsequent years fostering more independence. Most interviewees agreed that residents could be clinically independent after 2 years but thought a 3-year residency would be a net benefit to rural clinics and may increase retention.

### Developing financial support

Interviewees generally thought that a rural naturopathic residency would need to be funded like other rural residency programs, using multiple income streams. Administrators thought that insurance reimbursements, grants, and sponsorships from academic or professional institutions would provide adequate support. Regarding ND services being reimbursable by Medicaid and most private insurances, one interviewee stated that “Once you are over the hump, ND residents should be revenue generators comparable to anyone else.”

While most federal grants do not cover naturopathic services, one site noted the Health Resources and Services Administration (HRSA)'s Substance Abuse Expansion Service as an exception. State programs such as loan repayment in Oregon and Washington were more inclusive of NDs working with underserved populations; some administrators regarded these grants as a largely untapped resource to support NDs in rural primary care.

Interviewees were unanimous in reporting that the lack of Medicare reimbursement for ND services was a significant barrier to developing residencies. One site employed its ND as an in-house “safety net” provider for acute office visits with Medicare enrollees when other providers were unavailable; the cost of these visits had to be recouped through grants. Other sites had strict policies preventing NDs from seeing Medicare patients unless they paid out of pocket, which some patients were willing to do. Patients of the ND at this site were said to “graduate out of [ND's] office” once they turned 65: “they cannot pay for any services, but they want to. … It's not fair, but it is what it is.” One administrator had lobbied their federal representatives for Medicare coverage of naturopathic medicine.

### Promoting naturopathic primary care to rural FQHCs

Study interviewees exhibited a spectrum of awareness about naturopathic medicine. Administrators from the sites that employed NDs had full knowledge of NDs' scope of practice and expressed positive feedback about their performance as PCPs. One administrator commented that the decision to hire NDs was driven by patient advocacy. Other administrators expressed a desire to hire providers such as NDs with an integrative medicine skillset for managing pain and other chronic conditions. As one rural administrator said, they wanted providers who can “better manage chronic disease, as the broad concepts of whole person health, social determinants of health, mind-body medicine, and adverse childhood experiences become a bigger part of our narrative about ‘health’ vs. ‘healthcare.’”

Interviewees that were less familiar with NDs expressed varying perceptions about naturopathic medical training and scope of practice. One rural administrator was unaware that NDs in Oregon and Washington could prescribe pharmaceuticals. Another administrator from a rural site without NDs associated them with the stereotype of “concierge” medicine, which conflicted with the mission of a FQHC to see more publicly insured patients with complex health conditions. In this respect, these interviewees doubted the fitness of naturopathic medical education for rural primary care.

Most interviewees recognized that conventional medical providers would have to “buy-in” to working with NDs, which might be easier at sites with leadership that embraces complementary and integrative medicine. One administrator cited “your old-school MDs” as the greatest obstacle to incorporating NDs at their center: “We probably lost a hospitalist over the issue.” The most common recommendation was for NDs to continue building interprofessional relationships in health care and demonstrate their skillset in the primary care setting. Several interviewees hypothesized that more exposure to NDs in rural health would inspire other clinics to create residency training opportunities for NDs.

## Discussion

This study is the first to explore the potential expansion of naturopathic residencies into the rural primary care setting. Further, no previous studies to our knowledge have described the current roles NDs serve in rural primary care. Some of our findings were consistent with previous literature describing the successes and challenges of existing rural residency programs for MDs/DOs. For example, other studies have identified mentorship as being difficult for some rural clinics to sustain due to a scarcity of resources.^23,24^

The sustainability of rural residency programs is often reliant on devoted mentors who are passionate about clinical education and who are strong leaders in their communities.^25,26^ This could be even more true for a naturopathic residency program, which would require strong advocacy from existing clinical leadership to become established in a community with little experience of NDs.

Our study findings suggest that residencies for NDs may be worth pursuing for some rural FQHCs in states where NDs have a broad scope of practice—examples include Vermont, Arizona, New Mexico, and Idaho, in addition to Oregon and Washington. Developing naturopathic residencies in community health centers could look similar to the development of nurse practitioner residencies, which began in a FQHC in 2007.^27,28^

In most states, NDs are similar to nurse practitioners in their eligibility for licensure and independent practice without residency training. However, nurse practitioners developed residencies in response to how underprepared new graduates felt when faced with the complex needs of underserved patients. Most NDs interviewed reported a similar perception of insufficient preparation and strongly advocated for more postgraduate clinical training.

The mutual benefits of rural residency training for providers and clinics are well known, including the perception of a more valuable learning experience compared with urban residencies and higher provider retention rates.^23,25,26^ These same benefits may also apply to a rural naturopathic residency program. Participating administrators from sites that already had NDs thought that the NDs successfully provided primary care and were well integrated in their clinics.

Interviewees stated unanimously that the lack of Medicare reimbursement for ND services was a significant barrier. Oregon and Washington are unique in that some Medicare Advantage plans cover naturopathic services, but these are less utilized in rural areas.^29^ State differences in licensure are also important to consider. For example, NDs in Oregon can prescribe all Schedule II drugs, whereas Washington NDs may only prescribe certain ones such as formulations of testosterone and codeine. Consequently, NDs in Washington are more limited in their ability to work with opioid-dependent patients; nevertheless, interviewees in both states generally favored the use of naturopathic alternatives for chronic pain patients.

Our study was limited by a relatively small convenience sample of clinics in Oregon and Washington, and more of these were in Washington. Further work would be needed to determine the applicability of our findings to other regions that have a different licensure and insurance landscape for NDs. Some results may be relevant to NDs in Canada due to its oversight by the same educational accrediting body (CNME). We opted for written field notes in lieu of audio recording interviews for the sake of expediency, but this was also a limitation since notetaking can interfere with follow-up questions that may reveal more themes and subthemes.

Due to the study's exploratory nature, we did not achieve saturation or try to validate our findings through member checking or other means. As with other qualitative research, ours was subject to participant bias. Strengths include the variety of communities and the variety of positions included.

We recommend that future studies examine patient health outcomes, health care expenditure, and health care utilization associated with naturopathic primary care in the rural setting. Future qualitative studies could examine rural community patients' and providers' perceptions of naturopathic medicine. Perspectives from the wider naturopathic profession on rural primary care residencies are also important to assess with regard to the feasibility of such programs.

## Conclusions

Awareness is mixed among rural FQHCs about the training and scope of practice that enable NDs to work in this setting. The ND residencies in rural FQHCs may need to be financially supported by a combination of naturopathic institutions and public/private grants. The ND residents will need strong on-site mentors and opportunities for continuing education specific to rural health care needs, such as emergency medicine. With appropriate program funding and support, rural residency training for NDs may provide a mutual benefit to ND residents and rural communities in need of PCPs.

## Notes

**Cite this article as:** Garofalo L, Bell T, Poling G, Patterson D (2023) Assessing the feasibility of rural residency training for licensed naturopathic physicians in the northwest: a qualitative study, *Integrative Medicine Reports* 2:1, 43–50, DOI: 10.1089/imr.2022.0089.

## Abbreviations Used

BHW: Bureau of Health Workforce
CNME: Council on Naturopathic Medical Education
FQHCs: federally qualified health centers
HHS: U.S. Department of Health and Human Services
HRSA: Health Resources and Services Administration
MD/DO: Doctor of Medicine/Doctor of Osteopathy
NCCIH: National Center for Complementary and Integrative Health
ND: naturopathic physicians
PCPs: primary care providers
